# The Impact of Migration Status on Adolescents’ Mental Health during COVID-19

**DOI:** 10.3390/healthcare10010176

**Published:** 2022-01-17

**Authors:** Christoph Pieh, Rachel Dale, Andrea Jesser, Thomas Probst, Paul L. Plener, Elke Humer

**Affiliations:** 1 Department for Psychotherapy and Biopsychosocial Health, Danube University Krems, 3500 Krems, Austria; christoph.pieh@donau-uni.ac.at (C.P.); rachel.dale@donau-uni.ac.at (R.D.); andrea.jesser@donau-uni.ac.at (A.J.); thomas.probst@donau-uni.ac.at (T.P.); 2 Department of Child and Adolescent Psychiatry, Medical University of Vienna, 1090 Vienna, Austria; paul.plener@meduniwien.ac.at; 3 Department of Child and Adolescent Psychiatry and Psychotherapy, University of Ulm, 89081 Ulm, Germany

**Keywords:** migration status, adolescents, mental health, COVID-19, depression, anxiety

## Abstract

The purpose of this study was to compare mental health in adolescents with and without migration background after a semester of remote schooling and almost a year of social distancing in Austria. An online survey, supported by the Austrian Federal Ministry of Education, Science and Research, was conducted from 3rd February to 28th February 2021 measuring well-being (WHO-5), depression (PHQ-9), anxiety (GAD-7), sleep quality (ISI), stress (PSS-10), and disordered eating (EAT-8). A matched-pairs analysis with and without migration background was conducted and was checked with whole sample analysis. From a total of 3052 participants, N = 508 had a migration background (first or second generation) and N = 479 could be matched according to age, gender, region, and education with adolescents without migration background. Matched-pairs analyses showed that migration background is associated with poorer mental health concerning well-being, depression, anxiety, and insomnia scores (all *p*-values < 0.05). Prevalence of depressive symptoms (64.5% vs. 56.5%), anxiety symptoms (53.5% vs. 46.0%), as well as insomnia (31.9% vs. 21.0%) is higher in adolescents with migration background (all *p*-values ≤ 0.02). Comparison of the whole sample (N = 3052) confirmed these results. Results suggest that migration status is a risk factor for mental health problems among adolescents during the COVID-19 pandemic and highlight the need to implement easily accessible culture- and language-specific health promotion and prevention strategies.

## 1. Introduction

According to a recent report of the international Health Behavior in School-aged Children (HBSC) study, across all European countries, over 20% of adolescents have an immigration background [1]. Research on the association between adolescent mental health and immigration background is inconclusive [2,3]. Most studies show that the mental health of migrants is poorer [4,5], which is explained by several factors such as economic disadvantages, stressors during migration, acculturation stress, as well as heightened barriers to assess mental health services [6]. A meta-analysis from 51 studies and 224,197 participants found significantly worse mental health outcomes in immigrant children and youth, independently of generation [7]. A review of 36 studies confirmed that there is an increased risk of internalizing problems in young people with immigrant roots. The authors point out that there might be other relevant factors influencing immigrant adolescents’ mental health, such as low socioeconomic status, a non-European origin, or gender effects [8].

In contrast, Mood et al. [9] found fewer internalizing and externalizing problems in immigrants than in their native peers in England, Germany, the Netherlands, and Sweden. This phenomenon is referred to as the “immigrant health paradox” [10]. Arguments explaining such findings point out that immigrant youth might share protective factors supporting resilience [11], such as a stronger sense of family cohesion, obligation, and academic motivation—all attributes that are positively related to psychological well-being [4,12].

The COVID-19 situation and associated events, such as lockdown measures and school closures, had considerable negative effects on the mental well-being of adolescents internationally, as several recent studies and reviews have shown [13,14,15,16,17]. In Austria, Pieh et al. [18] found a significant decrease in mental well-being as well as a high prevalence of mental disorders in adolescents one year after the COVID-19 outbreak. In a comprehensive systematic review, Gibson et al. [19] assessed the impact of various inequality factors on mental health outcomes during the COVID-19 pandemic, implying that health inequalities in society may be further exacerbated by the current crisis. The authors emphasized the necessity of an intersectionality perspective, pointing out that interacting inequality factors may produce a higher impact on mental health outcomes than one inequality factor alone. One of their results was that both age and immigrant background generally had a negative effect on mental health. However, the evidence base was scarce as only 10 studies were found investigating the effect of immigrant background on mental health and the respective authors did not discuss results in depth. In contrast to the results of the review, Liu et al. [20] found that compared to the native U.S. population, young adults (18–30 years) with an Asian or Hispanic background reported lower levels of mental health symptoms during the COVID-19 pandemic. A study by Akkaya-Kalayci et al. [21], on the other hand, found no difference in mental health outcomes between native Austrian and immigrant Turkish youth aged 15–25, although adolescents of immigrant origin were significantly more fearful than their native peers that their family members could get infected with COVID-19.

It can be expected that the existing vulnerabilities of immigrant youth are increased by the pandemic. Like many other countries, Austria imposed lockdown measures of several degrees to reduce COVID-19 transmission. In addition to curfews, the Austrian government took the precaution of closing schools to contain the spread of the virus at different time points. From October 2020 to February 2021, all schoolchildren aged 14 or higher received almost exclusively remote schooling in Austria. The current study aimed to assess potential differences in mental health in adolescent students with or without migration background in Austria after several months of almost exclusive remote schooling.

## 2. Materials and Methods

### 2.1. Study Design

This cross-sectional online study was conducted from 3rd February to 28th February 2021 in Austria. It was supported by the Austrian Federal Ministry of Education, Science and Research. The Federal Ministry informed all high schools and asked them to forward the link to their students. The study was carried out via Research Electronic Data Capture (REDCap) [22]; REDCap is hosted on the servers of the Danube-University Krems, Austria. All participating adolescents had to agree to the data protection declaration to start the survey (electronic informed consent) and had to confirm that they were aged 14 or older. The principles outlined in the Declaration of Helsinki were followed, and the ethics committee of the Danube-University Krems as well as the data protection officer of the Danube-University Krems approved the study (EK GZ 41/2018–2021).

### 2.2. Migration Status, Restrictions, and Remote Schooling in Austria

To assess the migration status, students were first asked whether both parents were born abroad, following the definition of second-generation immigrants. They were further asked whether they were born abroad, to assess the number of first-generation immigrants. Participants who answered yes to one or both of these questions were considered to have a migration background.

From October 2020 until the time of the study, secondary school took place mainly via remote schooling. In addition, during this time, no team or indoor sports, as well as no public gatherings of children or adolescents were allowed. Various social distancing measures have been in place since March 2020. Since then, three strict lockdowns (from 16 March to 30 April 2020, from 17 November to 6 December 2020, and from 26 December 2020 to 7 February 2021) have been imposed. During the strict lockdowns, one was allowed to leave the household only in a few situations. On 8 February 2021, the strict lockdown was lifted and secondary schools were allowed to reopen with classes in a shift system and with extended protective measures such as weekly COVID-19 tests and wearing FFP2 face masks.

### 2.3. Measures

The following measures were applied to assess mental health. These measures were selected because they are validated in German and in adolescents and are widely used in the research literature for assessing mental health and symptoms.

#### 2.3.1. Well-Being (WHO-5)

The WHO-5 [23] is a self-rating questionnaire for well-being, comprising five items rated on a six-point Likert scale. The total score can range from 0 (absence of well-being) to 100 (maximal well-being). The German version of the WHO-5 has been validated in adolescents [24] and was used in the current study. In the current sample, Cronbach’s alpha for well-being was α = 0.85.

#### 2.3.2. Depressive Symptoms (PHQ-9)

Depressive symptoms were measured with the depression module of the Patient Health Questionnaire (PHQ-9) [25]. The PHQ-9 comprises nine items that are self-rated on a four-point scale ranging from 0 (“not at all”) to 3 (“nearly every day”), resulting in a sum score ranging from 0 to 27. The German version of the PHQ-9 is validated for adolescents [26]. In the current study, we used a cut-off score of ≥11 points to define clinically relevant depression, as this cut-off is recommended for detecting major depression in adolescents [27]. Cronbach’s alpha for depression was α = 0.88 in our study sample.

Item 9 of the PHQ-9 was used as an indicator of suicidal ideation [28]. Item 9 of the PHQ-9 asks “Over the last two weeks, how often have you been bothered by thoughts that you would be better off dead or of hurting yourself in some way?”. Response to this question was coded in a binary way to detect any suicidal ideas within the last two weeks (presence of suicidal thoughts = response to item 9 ranged from 1 to 3; absence of suicidal thoughts = response to item 9 was 0).

#### 2.3.3. Anxiety Symptoms (GAD-7)

Anxiety symptoms were measured with the German version of the Generalized Anxiety Disorder 7 scale (GAD-7) [29]. The GAD-7 comprises seven self-rated items on a four-point scale from 0 to 3, resulting in a sum score ranging from 0 to 21. A cut-off point of ≥11 for moderate symptom levels was suggested for adolescents [30] and therefore used in the current study to define clinically relevant anxiety. Cronbach’s alpha was α = 0.88.

#### 2.3.4. Sleep Quality (ISI)

Sleep quality and insomnia were measured with the German version of the Insomnia Severity Index (ISI), which is validated in adolescents [31]. The ISI comprises seven self-reported items on a four-point scale, from 0 to 4 (maximum score 28). A cut-off score of ≥15 points is recommended for clinical insomnia (moderate severity) [32]. Cronbach’s alpha for sleep quality was α = 0.80.

#### 2.3.5. Stress (PSS-10)

The Perceived Stress Scale (PSS-10) is a validated and widely used 10-item questionnaire to measure stress [33]. For the last month, the stress level is rated on five-point scales ranging from 0 to 4 (maximum score 40), with a higher score indicating higher perceived stress, with scores ≥27 indicating high stress. The German version is validated for adolescents aged 14 years or older [34]. Cronbach’s alpha was α = 0.87 in the current sample.

#### 2.3.6. Eating Attitudes Test (EAT-8)

Disordered eating was measured with the EAT-8[35], a validated instrument to screen for individuals at high risk of developing clinical eating disorders. The EAT-8 includes eight self-rated items in a dichotomized response format (1 = “I agree somewhat” and 0 = “I disagree somewhat”), resulting in a sum score ranging from 0 to 8. Respondents can be classified into either a low-risk or a high-risk group. In the current study, the recommended cut-off points of 3 for female and 2 for male adolescents were used [35]. Cronbach’s alpha for disordered eating was α = 0.84.

### 2.4. Statistical Analyses

Two independent statistical methods were performed. Firstly, a matched pair analysis, and secondly, a comparison of the total sample regarding migration status. The significance level was set at 0.05, and all statistical tests were performed two-tailed. Migration generation (first or second) was not accounted for in the statistical analyses, as first-generation and second-generation immigrants did not differ in any of the analyzed outcome variables (all *p*-values > 0.05).

#### 2.4.1. Matched-Pair Analysis

Data were analyzed using R version 4.0.3 (R Foundation, Vienna, Austria) for the matched-pairs analysis [36]. A matched-pairs analysis according to age, gender, region, and education type was conducted using the MatchIt package [37]. Matching was conducted using the nearest neighbor method to match only one respondent with no migration background to each respondent with a migration background. A caliper of 0.1 was implemented to ensure that only pairs that matched closely were included. Age and gender were included in the matching as they are standard variables where mental health differences are found. School type and region were found to significantly differ between the two groups and were therefore also included (school: χ2 = 25.02, *p* < 0.001; region: χ2 = 115.36, *p* < 0.001). After matching, the two groups were compared in the mental health outcome measures using linear models with migration background and propensity score as fixed factors. For assessing the likelihood of being over the cut-offs, general linear models were used with the binomial family.

#### 2.4.2. Whole Sample Analysis

Whole sample analyses were conducted with the IBM SPSS Statistics software (IBM Corporation, Armonk, NY, USA) version 26 [38]. T-tests for independent samples were computed to assess potential differences between adolescents with migration status and those with no migration status in the mean values of each variable. Chi-squared tests were computed to analyze differences between migration vs. non-migration status in the percentage of individuals scoring above the cut-offs for depression, anxiety, insomnia, high stress, and disordered eating.

## 3. Results

A total of N = 3052 (70.1.% females) adolescents participated in the study. From all, N = 508, participants with a migration background (first or second generation), N = 479 could be matched according to age, gender, region, and education. Sociodemographic characteristics of the study sample are summarized in Table 1.

### 3.1. Matched-Pairs Analysis (N = 958)

In total, N = 479 adolescents with a migration background (first and second generation) could be matched according to age, gender, region, and education. In total, 64.5% of adolescents with migration background scored above the cut-off for depression (PHQ-9 ≥ 11) compared to 56.5% of those without migration background (*p* = 0.01; Table 2). In addition, anxiety symptoms (GAD-7 ≥ 11) were more prevalent (53.5% vs. 46%) in adolescents with migration status (*p* = 0.02). Similarly, a higher prevalence of insomnia (ISI ≥ 15) was observed in adolescents with a migration background as compared to those without (31.9% vs. 21%; *p* < 0.001). The prevalence of suicidal ideation as assessed by a single item from the PHQ-9 was higher in students with migration status (43.4%) as compared to those without (35.5%; z(883) = 2.58; *p* < 0.01). In total, 19.8% of adolescents with migration status reported suicidal ideation “almost daily” or “more than every other day” compared to 16.1% of those without migration background z(883) = 1.53; *p* = 0.13); 11.5% adolescents with migration background reported suicidal ideation “almost daily” vs. 7.9% in those without migration background (z(883) = 1.86; *p* = 0.06).

In addition, mean scores of well-being (WHO-5), depressive symptoms (PHQ-9), anxiety symptoms (GAD-7), and insomnia (ISI) differed according to migration status (all *p*-values < 0.05; Table 3). Stress-level and disordered eating did not differ according to mean scores or cut-offs (Table 2 and Table 3).

### 3.2. Whole Sample Analysis (N = 3052)

To verify the results of the matched-pairs analysis, whole sample analysis was conducted with similar results. Adolescents with a migration background showed a higher prevalence of depressive symptoms, anxiety symptoms, and clinical insomnia (*p*-values ≤ 0.002; Table 4 and Table 5). Furthermore, significant differences were found in the prevalence of highly stressed adolescents and mean PSS-scores (*p* ≤ 0.002). The prevalence of suicidal ideation was higher in students with migration status (47.3%) as compared to those without (34.9%; χ^2^(1; 2755) = 24.68; *p* < 0.001). In total, 21.6% of adolescents with migration status reported suicidal ideation “almost daily” or “more than every other day” compared to 15.3% of those without migration background χ^2^(1; 2755) = 10.998; *p* = 0.001); 12.6% adolescents with migration background reported suicidal ideation “almost daily” vs. 8.2% in those without migration background (χ^2^(1; 2755) = 8.831; *p* = 0.003).

## 4. Discussion

Adolescents with first- or second-generation migration backgrounds are more likely to have psychological symptoms compared to those without during COVID-19. Almost two-thirds of adolescents with migration background suffer from at least moderate depressive symptoms, over half suffer from anxiety symptoms, and about one-third from clinical insomnia. Moreover, suicidal ideations are more prevalent in adolescents with migration backgrounds. Eating disorders did not differ between adolescents with migration background and those without.

In accordance with our results, in a representative study in Germany conducted between May and June 2020, in which two-thirds of N = 1040 11- to 17-year-old children and adolescents reported being highly burdened, children with migration background were affected significantly more [39]. A longitudinal study conducted in adolescents aged 16–19 before the COVID-19 pandemic (2018/2019) and during the first COVID-19 lockdown (May to July 2020) in Germany observed a stronger increase in the prevalence of depressive symptoms in adolescents with a migration background (from 11% to 33%) compared to those without (from 9% to 21%) [40]. In our sample, 43.4% of adolescents with a migration background reported suicidal ideation several days within the past two weeks compared to 35.5% in adolescents without migration background. A European study before COVID-19 reported an elevated risk of suicidality within the last two weeks in 4.9% of adolescent participants [41]. To our knowledge, there are no studies available on adolescent suicidality during the pandemic; however, a preliminary study conducted in the US revealed a potential association of COVID-19-related fears and confinement measures with suicidal thoughts and attempts in adults [42].

There are several explanations for the disadvantage regarding the mental health of adolescents with a migration background. Burdens for mental health can stem from stressful and potentially traumatic events in former home countries, the process of flight, as well as difficulties with re-settling in a foreign country. Acculturation theory stresses that major life changes brought about by leaving behind family and friends, customs, and familiar surroundings and adjusting to a new cultural environment with different norms and values and a new language can cause psychological distress [3]. Theories about social stress refer to shared experiences such as economic hardship, social exclusion, and discrimination causing negative effects on adolescent mental health [2,5]. Having immigrant roots entails being in the minority in the host country, which may result in feelings of isolation and, consequently, psychological distress [8]. In general, children and young people find it easier than adults to familiarize themselves with a foreign culture and adapt to the customs of a new country. They can adapt to the language and culture of the host country more quickly, which can lead to generational conflicts [43].

Independent of migration, adolescence is a sensitive period of social development with social interactions becoming increasingly important [44]. Adolescence involves numerous profound biopsychosocial transformations and psychological and social challenges [39]. Compared with children, adolescents spend less time with their family, while they form more complex peer relationships [45]. This facilitates gaining independence from parents and enables them to explore various domains of identity and to foster a more complete sense of social self-identity [46]. High-quality peer relationships are also important in protecting against mental health problems, as adolescence is well known as a period of enhanced vulnerability to mental health disorders [47]. Therefore, coping with the current COVID-19 situation and complying with the restrictions, such as enforced physical distancing and reduced in-person contact with peers, might substantially impair psychological development in adolescents since these conditions can be experienced as being incongruent with their developmental tasks [39,45].

In general, income levels are lower in people with immigrant backgrounds in Austria [48], which entails poorer living conditions and a lower socioeconomic status as compared to the majority of the population [2]. During the curfews and periods of remote schooling, immigrant families might have been less able to provide their children with the necessary equipment, access to online learning, or a private individual learning space [21]. Due to language barriers, parents might have been less able to support their children with school affairs and learning. In addition, immigrant families might have suffered more from economic and financial constraints due to the crisis (e.g., job loss), which might result in increased levels of stress [49]. Moreover, young people with an immigrant background are known to have more difficulties accessing health care services [5,50], which might further worsen existing mental conditions during the pandemic.

This study has several limitations. First, the cross-sectional nature of the study allows no causal conclusions as to whether the COVID-19 situation further aggravated mental health issues in adolescents with migration background or whether higher mental health burden in adolescents with migration status rather reflected pre-existing differences in mental health. Thus, an additional survey before the COVID-19 pandemic would have assisted to study changes in mental health in youth with vs. without migration background adequately. A further limitation is that no information on the socioeconomic status was obtained; therefore, it was not possible to elucidate the extent to which the poorer mental health in students with migration background is associated with migratory status and not with poorer socioeconomic status. Furthermore, the study design did not allow for analyses stratified by specific immigrant group characteristics. As migration background in Europe is not, in general, a predictor for higher behavioral or emotional problems [8], potential differences among immigrant groups are required. As a few examples, the country of origin, reasons for immigration, gender, acculturation style [51], attitudes towards immigrants among the majority of the population [9], immigrant policies [7], and social contexts—e.g., school culture—within which adolescents are living [4] should be analyzed in future studies. Although individuals with migration background and those without migration background did not differ with respect to gender and age, they differed with respect to region and school type. Therefore, the matched-pairs analyses were conducted on a sample matched according to age, gender, region, and education type. Statistical analysis conducted on the total samples largely confirmed results obtained from analyses of matched pairs.

## 5. Conclusions

Overall, this study suggests that migration background is a risk factor for mental health problems among adolescents during the COVID-19 pandemic. Students with migration status seem to be particularly burdened mentally by the COVID-19 pandemic and associated confinement measures such as remote schooling, which might significantly contribute to increased inequalities. Therefore, it is crucial to react to the increased mental health burden by allowing time for re-settlement in the school context and reducing stressors in schools. Attention should be paid to adolescents at high risk to suffer from negative consequences of the COVID-19 pandemic and associated confinement measures on mental health, to reduce health inequalities.

Adolescence is a phase of life in which behavioral patterns are shaped for the future and the course is set for a long and healthy life. For this reason, and also given the high prevalence of mental health disorders and stress, there is a need to identify incipient mental problems at an early stage and to counter them preventively. As difficulties accessing health care services and low mental health literacy are common in young people with migration backgrounds [5,50], it is of high importance to facilitate access of youth with migration backgrounds to low-threshold mental health care services. Thus, there is a high need to initiate nationwide targeted, low-threshold preventive measures, especially for vulnerable and marginalized populations, such as adolescents with migration background. Next to the offer of culture- and language-sensitive mental health services for this population, diversity-care measures are of utmost importance, and professionals working in the health care system should be trained to gain transcultural competence for treating migrants adequately.

Further studies are needed to better understand mental health needs and potential barriers to mental health care services among immigrant populations.

## Figures and Tables

**Table 1:** Matched sample characteristics (N = 958).

Variable	Migration Background (N)	No Migration Background (N)
Total	479	479
Gender		
Female	333	342
Male	142	134
Diverse	4	3
Migration		
Parents born abroad	312	-
Both parents and self born abroad	133	-
Only self born abroad	34	-
Age		
14	42	41
15	92	96
16	110	108
17	119	118
18	85	87
19–20	31	29
Region		
North-East (Vienna, Lower Austria, Upper Austria)	296	293
South-East (Carinthia, Styria, Burgenland)	65	69
West (Tyrol, Salzburg, Vorarlberg)	118	117
School		
Middle school	5	3
Polytechnical School	2	2
Part-Time Vocational School and Apprenticeship (Dual Training)	17	14
School for Intermediate Vocational Education	19	19
College for Higher Vocational Education	194	193
Academic secondary school	242	247
Other	-	1

**Table 2:** Prevalence of moderate depressive symptoms, anxiety symptoms, clinical insomnia, eating symptoms, and high stress by migration status (matched pairs N = 958).

		Migration Background	No Migration Background	Statistics
PHQ-9 score	<11	157 (35.5)	192 (43.5)	z(882) = 2.44
N (%)	≥11	285 (64.5)	249 (56.5)	*p* = 0.01
GAD-7 score	<11	210 (46.5)	247 (54.0)	z(908) = 2.28
N (%)	≥11	242 (53.5)	210 (46.0)	*p* = 0.02
ISI score	<15	318 (68.1)	376 (79.0)	z(942) = 3.77
N (%)	≥15	149 (31.9)	100 (21.0)	*p* < 0.001
EAT-8 score	<2/<3	167 (36.8)	187 (40.5)	z(915) = 1.15
N (%)	≥2/≥3	287 (63.2)	275 (59.5)	*p* = 0.25
PSS-10 score	<27	262 (57.0)	293 (62.9)	z(925) = 1.84
N (%)	≥27	198 (43.0)	173 (37.1)	*p* = 0.07

Note: *p*: *p*-values (2-tailed); ISI: Insomnia Severity Index; GAD-7: Generalized Anxiety Disorder 7 scale; PHQ-9: Patient Health Questionnaire 9 scale; PSS-10: Perceived Stress Scale 10; EAT-8: Eating Attitudes Test 8; an EAT-8 cut-off score of ≥2 in male and ≥3 in female and gender-diverse adolescents was considered as being indicative of disordered eating.

**Table 3:** Mean scores of psychological health by migration background (matched pairs N = 958).

		Migration Background	No Migration Background	Statistics
WHO-5	M	33.85	37.82	t(955) = −3.0; *p* < 0.01
	SD	20.74	20.32	
	N	479	479	
PHQ-9	M	13.37	11.94	t(880) = 3.22; *p* = 0.001
	SD	(6.59)	(6.60)	
	N	442	441	
GAD-7	M	11.04	10.34	t(906) = 1.98; *p* = 0.048
	SD	(5.29)	(5.25)	
	N	452	457	
PSS-10	M	24.35	23.74	t(923) = 1.29; *p* = 0.20
	SD	(6.79)	(7.50)	
	N	460	466	
ISI	M	11.84	10.14	t(940) = 4.72; *p* < 0.001
	SD	(5.55)	(5.49)	
	N	467	476	
EAT-8	M	3.53	3.49	t(919) = 0.17; *p* = 0.86
	SD	(2.62)	(2.70)	
	N	457	465	

Note: *p*: *p*-values (2-tailed); M: mean score; SD: standard deviation, t: t-test; ISI: Insomnia Severity Index; GAD-7: Generalized Anxiety Disorder 7 scale; PHQ-9: Patient Health Questionnaire 9 scale; PSS-10: Perceived Stress Scale 10; WHO-5: Well-being questionnaire of the World Health Organization (WHO); EAT-8: Eating Attitudes Test 8.

**Table 4:** Prevalence of moderate depressive symptoms, anxiety symptoms, clinical insomnia, eating symptoms, and high stress by migration status (whole sample, N = 3052).

		Migration Background	No Migration Background	Statistics
PHQ-9 score	<11	157 (35.4)	1081 (46.8)	χ^2^(1;2752) = 19.82
N (%)	≥11	287 (64.6)	1227 (53.2)	*p* < 0.001
GAD-7 score	<11	211 (46.5)	1284 (54.2)	χ ^2^(1;2821) = 9.23
N (%)	≥11	243 (53.5)	1083 (45.8)	*p* = 0.002
ISI score	<15	332 (68.0)	1976 (79.0)	χ ^2^(1;2988) = 28.14
N (%)	≥15	156 (32.0)	524 (21.0)	*p* < 0.001
EAT-8 score	<2/<3	169 (36.7)	991 (41.3)	χ ^2^(1;2862) = 3.42
N (%)	≥2/≥3	292 (63.3)	1410 (58.7)	*p* = 0.065
PSS-10 score	<27	266 (57.2)	1564 (64.7)	χ ^2^(1;2881) = 9.54
N (%)	≥27	199 (42.8)	852 (35.3)	*p* = 0.002

Note: *p*-values (2-tailed); χ^2^: chi-square; ISI: Insomnia Severity Index; GAD-7: Generalized Anxiety Disorder 7 scale; PHQ-9: Patient Health Questionnaire 9 scale; PSS-10: Perceived Stress Scale 10; EAT-8: Eating Attitudes Test 8; an EAT-8 cut-off score of ≥ 2 in male and ≥3 in female and gender-diverse adolescents was considered as being indicative of disordered eating.

**Table 5:** Mean score of psychological health by migration background (whole sample, N = 3052).

		Migration Background	No Migration Background	Statistics
WHO-5	M	33.86	38.62	t(3047) = −4.71; *p* < 0.001
	SD	(20.71)	(20.80)	
	N	507	2542	
PHQ-9	M	11.39	11.61	t(2750) = 5.22; *p* <.001
	SD	(6.60)	(6.57)	
	N	444	2308	
GAD-7	M	11.06	10.18	t(2819) = 3.23; *p* < 0.001
	SD	(5.30)	(5.26)	
	N	454	2367	
PSS-10	M	24.34	23.34	t(705.2) = 2.87; *p* = 0.004
	SD	(6.80)	(7.59)	
	N	465	2416	
ISI	M	11.87	10.01	t(2986) = 6.71; *p* < 0.001
	SD	(5.53)	(5.62)	
	N	488	2500	
EAT-8	M	3.51	3.35	t(2860) = 1.217; *p* = 0.224
	SD	(2.62)	(2.66)	
	N	461	2401	

Note: *p*: *p*-values (2-tailed); M: mean score; SD: standard deviation, t: t-test; ISI: Insomnia Severity Index, GAD-7: Generalized Anxiety Disorder 7 scale; PHQ-9: Patient Health Questionnaire 9 scale; PSS-10: Perceived Stress Scale 10; WHO-5: Well-being questionnaire of the World Health Organization (WHO); EAT-8: Eating Attitudes Test 8.

## Data Availability

Data are available upon request.
